# Involving students in real-world research: a pilot study for teaching public health and research skills

**DOI:** 10.1186/1472-6920-9-45

**Published:** 2009-07-16

**Authors:** Elinor Millar, Michael G Baker, Philippa Howden-Chapman, Nick Wilson, Nigel Dickson

**Affiliations:** 1 Department of Public Health, University of Otago, Wellington, New Zealand; 2 Department of Social and Preventive Medicine, University of Otago, Dunedin, New Zealand

## Abstract

**Background:**

There is some evidence that medical students consider population health issues less important than other domains in the health sciences and attitudes to this field may become more negative as training progresses. A need to improve research skills among medical students has also been suggested. Therefore we piloted an integrative teaching exercise that combined teaching of research skills and public health, with real-world research.

**Methods:**

Third year medical students at the University of Otago (Dunedin, New Zealand) filled in a questionnaire on their housing conditions and health. The students were given the results of the survey to discuss in a subsequent class. Student response to this teaching exercise was assessed using a Course Evaluation Questionnaire.

**Results:**

Of the 210 students in the class, 136 completed the Course Evaluation Questionnaire (65%). A majority of those who responded (77%) greatly supported or supported the use of the survey and seminar discussion for future third year classes. Most (70%) thought that the session had made them more aware and concerned about societal problems, and 72% felt that they now had an improved understanding of the environmental determinants of health. Students liked the relevance and interaction of the session, but thought it could be improved by the inclusion of small group discussion. The findings of the students' housing and health were considered by the tutors to be of sufficient value to submit to a scientific journal and are now contributing to community action to improve student housing in the city.

**Conclusion:**

In this pilot study it was feasible to integrate medical student teaching with real-world research. A large majority of the students responded favourably to the teaching exercise and this was generally successful in raising the profile of public health and research. This approach to integrated teaching/research should be considered further in health sciences training and continue to be evaluated and refined.

## Background

There is some evidence that medical students consider that prevention and population health issues are a matter of common sense, and are not as important as other domains in the health sciences [1]. While first year students generally have a positive attitude towards prevention and health promotion [2], this attitude seems to decline as they progress through medical school [3-5]. This decline could be the result of negative role modelling as some authors [1] found that only 41% of second year medical students at an Australian medical school thought that there was a positive attitude towards psychosocial and preventive health issues in their school. This phenomenon has also been reported in New Zealand, with both medical students and teachers sceptical of the importance of population health [6].

In addition, many medical graduates feel they have insufficient knowledge when it comes to basic research skills [7]. This deficit could be due to the fact that students are often not taught these skills and do not value them in their under-graduate curriculum [4]. A low importance attached to public health, along with little understanding of research skills is not a promising combination, especially given the growing importance of evidence-based approaches to making health sector activities more effective and cost-effective.

Currently there is very little literature that presents innovative ways of teaching public health research methods to undergraduate medical students. To try and fill this perceived gap in medical education, we piloted a method of teaching public health and research skills whereby students were required to interpret the results from surveys they had filled in about their own housing and personal respiratory health. This article aims to consider the feasibility and perceived value of this integrated teaching/research approach.

## Methods

### Research topic

We selected the research topic of student housing and health because this was an issue that could be studied in class and was likely to be of interest to the students. It was also an area in which the lecturers had expertise, had a good possibility of generating interesting findings, and is an important public health problem. There is existing evidence that New Zealand has relatively poor housing quality [8,9], with notably high rates of reported dampness, mould or water damage [10-15]. Furthermore, there is evidence that these conditions are associated with respiratory symptoms in New Zealanders [16-18]. The two tutors had conducted two randomised community trials of installing insulation and improved heating, which showed a significant and cumulative impact on reducing respiratory symptoms [11,19]. They had also conducted a case-control study of crowding and infectious disease [20], and an observational study of increased injuries in houses with injury hazards [21]. These studies were used to illustrate the research evidence of the impact of housing on health, as well as implicitly establishing the credibility of the tutors as researchers.

Both of these intervention studies had been recently published in the *British Medical Journal *and had received considerable media attention in New Zealand. Nevertheless, there had been no published New Zealand research on the impact of poor housing on the health of students, despite the relatively cold climate of the world's most southern medical school in Dunedin, where the teaching was carried out.

### Search for existing research teaching approaches

There seems to be very limited literature on teaching research skills to medical students. Of the literature available the majority was based around individuals or groups of students completing research projects [22,23]. whereas others were optional summer programmes [24]. Whilst carrying out research is the ultimate way of learning research methods it is also essential students have a base knowledge. For this reason some courses offer a didactic component on research methods before students begin their research [22,24]. Currently medical students at the University of Otago complete a group literature search project in their last preclinical year (third year) as well as a research project during their public health rotation during their clinical years.

While there was very little literature about teaching research methods to medical students, there did seem to be more related to nursing. Research is often unpopular with nurses and nursing students, with the most common reason being a preference for practical work [25]. This situation may be hindering nurses from using the most up-to-date medical care approaches, as many feel they are poorly taught basic statistics and analytical skills so do not feel confident in their ability to evaluate the quality of research they read [26,27].

An important issue in research training seems to be timing. Some research found that students wanted research teaching at the beginning of their degree so that they could apply the skills they learn from it to their assignments throughout their course [28], whilst others felt that introducing a research module too early, before students were aware of its relevance, left students with a negative attitude towards research [25].

### Housing and Health Survey

A questionnaire on housing and health was developed by the public health course tutors and colleagues. As well as standard demographic information, the questionnaire included two main components: one on the housing conditions of the students, and one on their respiratory health.

The housing conditions component was based on sections of the Health Housing Index (HHI) questionnaire which had been developed to systematically record the conditions of a house that are most relevant to its health and safety. The HHI questionnaire had been previously pilot tested on 100 homes and found to have useful predictive value (people living in houses with more injury hazards had a significantly greater probability of having injuries resulting in medical consultations [21]). For this teaching study we extracted the questions that related to the internal air environment of the house (covering questions on damp, mould, insulation, ventilation, heating, and general house conditions). Students were asked to complete 35 questions (with multiple possible response options) relating to their living conditions. For example, the question on subjective 'dampness' feel of the house included response options such as "feels dry throughout"; "feels damp in places", and "feels damp throughout".

Furthermore, there were 11 respiratory health questions that were based on those used in the International Study of Asthma and Allergies in Childhood (ISAAC) study [29]. Of these the key question was "have you had wheezing or whistling in your chest at any time over the last month"?

The questionnaire was first used in March 2007 and a slightly revised version used as part of this present study in March 2008. A copy is available at: . Ethics approval was provided through the University of Otago ethics approval system.

In March 2008, third year medical students at the University of Otago completed this anonymous questionnaire in a tutorial. The responses were entered into a database and analysed by staff at the Departments of Public Health in Dunedin and Wellington. The tabulated results were presented back to the students two weeks later as part of an integrative lecture given by the second and third authors.

### Teaching

The teaching took place as part of the "Patient, Doctor and Society" (PDS) section of the pre-clinical curriculum. PDS includes teaching on ethics, preventive and social medicine, professional development and evidence-based medicine. Throughout the year students participate in integrative days that focus on clinical scenarios and wider issues within healthcare such as complementary medicine, eating disorders, child health and domestic violence. The integrative morning on housing and health was held in a lecture theatre and took approximately two hours. The lecture, entitled "The Built Environment and Health", was divided into two sections. Firstly, students were presented with a definition of the built environment and explanation of why housing is important to health. Wherever possible, locally conducted, published research was used both as evidence and to explain the key epidemiological concepts of population exposure and risk [8,20,30]. Students were then shown the results of the local community trials looking at the impact housing has on health [11,19]. Both the methodology and the results were discussed in an interactive session and the links between population health and clinical symptoms emphasised.

The second part of the session examined the results of the medical student housing conditions survey. Students were given the aggregate survey results, which included tables showing the characteristics of the sample, their housing conditions and the reported frequency of respiratory symptoms. Further tables showed the association between reported housing exposures and respiratory symptoms (presented as frequencies, percentages, relative risks and 95% confidence intervals with p-values).

Students were then presented with a series of questions to answer on the survey and results. They were given time to work through and discuss them with the people around them, and then they were examined as a class in a lecturer-led discussion. The questions covered basic epidemiological principles (eg, "What sort of study is this?" and "What is the prevalence of asthma in this population?") as well as questions about interpreting the results and looking for limitations of the study (eg, "What hazards are associated with respiratory symptoms in this population?" and "What modifications could be made to this study to reduce the potential impact of reporting bias?"). There were also questions on the public health implications of the results (eg, "Do these findings support any housing interventions that might reduce the prevalence of respiratory symptoms in occupants?" and "Are there any wider public health implications from these findings?").

### Feedback

At the end of the teaching session students filled in an anonymous Course Evaluation Questionnaire. This process followed a standard method managed by the University's Higher Education Development Centre (HEDC). It used 10 questions, some of which were adapted from a catalogue of questions provided by HEDC (see Table 1). Students were asked to rate the value of the session on a Likert scale. They were also invited to add further comments about what they liked most and least about this form of teaching, and ways the teaching could be improved in the future.

**Table 1:** Results of medical student feedback on the value of a public health and research teaching exercise (covering their housing conditions and respiratory health, n = 136)

**Question**	**Likert Scale**						
How valuable has the student housing conditions survey, and discussion of findings, been for you?	**Extremely valuable**	**1**	**2**	**3**	**4**	**5**	**Not at all valuable**
	
	No.	29	60	32	12	3	
	%	21%	44%	24%	9%	2%	

Did this survey and seminar discussion improve your understanding of research methods?	**Yes, greatly**	**1**	**2**	**3**	**4**	**5**	**No, not at all**
	
	No.	18	52	47	13	6	
	%	13%	38%	35%	10%	4%	

Did this survey and seminar discussion improve your understanding of epidemiology?	**Yes, greatly**	**1**	**2**	**3**	**4**	**5**	**No, not at all**
	
	No.	16	41	52	20	7	
	%	12%	30%	38%	15%	5%	

Did this survey and seminar discussion improve your understanding of the environmental determinants of health?	**Yes, greatly**	**1**	**2**	**3**	**4**	**5**	**No, not at all**
	
	No.	28	69	30	7	2	
	%	21%	51%	22%	5%	1%	

Did this survey and seminar discussion improve your interest in public health?	**Yes, greatly**	**1**	**2**	**3**	**4**	**5**	**No, not at all**
	
	No.	23	58	34	12	9	
	%	17%	43%	25%	9%	7%	

Did this survey and seminar discussion improve your interest in health research?	**Yes, greatly**	**1**	**2**	**3**	**4**	**5**	**No, not at all**
	
	No.	16	48	46	15	11	
	%	12%	35%	34%	11%	8%	

Did this survey stimulate you to discuss housing and health issues with friends outside of class?	**Yes, often**	**1**	**2**	**3**	**4**	**5**	**No, never**
	
	No.	30	45	43	13	5	
	%	22%	33%	32%	10%	4%	

How much did this survey and seminar discussion challenge you to think?	**A great deal**	**1**	**2**	**3**	**4**	**5**	**Very Little**
	
	No.	21	63	35	11	5	
	%	15%	46%	26%	8%	4%	

Has this survey and seminar discussion made you more aware and concerned about societal problems?	**Yes, greatly**	**1**	**2**	**3**	**4**	**5**	**No, not at all**
	
	No.	38	57	32	4	4	
	%	28%	42%	24%	3%	3%	

Would you support use of this survey and seminar discussion for future 3^rd ^year classes?	**Yes, greatly**	**1**	**2**	**3**	**4**	**5**	**No, not at all**
	
	No.	56	49	23	6	2	
	%	41%	36%	17%	4%	1%	

## Results

### Quantitative assessment

Of the 210 students in the third year class, 136 filled in the Course Evaluation Questionnaire, a response rate of 65%. Overall the response from the feedback questionnaires were positive, with 77% greatly supporting or supporting (scores "1" or "2" in Table 1) the use of the survey and seminar discussion for future third year classes. Only 8 students (6%) would not support the session being repeated (17% were indifferent).

A majority of students (70%) thought that the session had made them more aware and concerned about societal problems, and 72% felt that they now had an improved understanding of the environmental determinants of health. Whilst 60% said the survey and discussion improved their interest in public health (25% neutral, 15% had no improved interest) only 47% felt that it improved their interest in health research (34% neutral and 19% gave scores "4" or "5"). Just over half (55%) of students reported the survey stimulated them to discuss housing and health issues with friends outside class.

### Qualitative assessment

The additional written feedback on the questionnaire was largely compatible with the Likert scale scores. In terms of things that students liked most about the teaching, 26 students (of the 69 who wrote additional comments) commented that they liked the interactive nature of the teaching: "interactive, more involved, more interesting"; "enabled us to experience how a cross-sectional survey is carried out"; and "we got to contribute."

Around a third of those who commented (n = 24) wrote that the teaching was a success because it was relevant and concerned the students directly: "it relates to us, applies to our housing standards"; "it relates to me! Cool!"; and "it concerns me and my own health".

Furthermore, when asked: "how could this form of teaching be improved in the future?", three main themes emerged. The first was that of time management: "condensing it down a bit"; "slightly more organised in terms of time schedules"; and "streamline it, make it shorter". Many students also felt it would have been more effective if there were small group tutorials instead of, or supplementing, the whole class discussion: "very useful, but [the] question section should be worked on in small groups"; "small group discussion versus lecture-based discussions"; and "smaller groups tutorials before whole class discussion."

The survey was carried out in March, near the beginning of the academic year (and early autumn in New Zealand). This timing meant many students had only been in their flats for a month and only knew what their flats were like in the warmer summer period. For this reason many students commented that they thought the survey should be carried out later in the year, when they thought the results may be more accurate.

## Discussion

This is the first evaluation study we are aware of that has reported on the use of an interactive research study to develop medical students' public health and research skills. It was practical to combine the research and teaching and a majority of the students responded favourably to the exercise. The class was generally successful in raising the profile of public health and research, but still was restricted by the built environment of the tiered lecture theatre which makes small group work difficult.

The poor quality of student flats in Dunedin has been a topic of considerable public interest and has resulted in a report presented to the media that found that some student flats were colder than a refrigerator during winter [15] as well as other unfavourable media reports [31]. The results of the student housing and health research have been written up and are in the process of being submitted to a journal (Baker M, Wilson N, Dickson N, Zhang J, Keall M, Crane J, Howden-Chapman P: Medical students have respiratory symptoms associated with poor housing conditions; draft manuscript). The findings were of intrinsic interest to the students and by raising the issue of their exposure to cold indoor temperatures in their accommodation, may have implications for protecting their health.

Evaluating a teaching session using a simple self-completed questionnaire distributed to a large class of medical students at the end of an extended teaching session has important limitations. The response rate of 65% could have been higher and it is possible that those who chose to respond may have differed from the non-responders in important ways (for example, by being more motivated by a desire to record positive or negative views about the teaching experience). This context also means that students inevitably had to provide an immediate and potentially rushed assessment of the teaching. Given those constraints it is surprising that so many students (51%) took the time to write additional comments on the questionnaire.

It is important to also note the potential issues that arise from this form of teaching. In this exercise the students effectively become the research subjects. This raises issues to do with informed consent and whether students felt that they could reasonably opt out of the research. This could become more of a problem if the approach was extended to other forms of health sciences training.

These results support continuation of this type of teaching/research combination, ideally with a switch to use of smaller groups for discussion of the findings. A greater emphasis on small group work is part of the current strategy for change in undergraduate teaching at the University of Otago. This type of teaching is very labour intensive as it requires rapid entry and analysis of questionnaire data and modification of presentation content to incorporate the results. It is therefore important that such teaching is evaluated to ensure its value to students.

If research methods and public health are to be seen as an integral part of the medical curriculum it needs to be integrated into the course. In 2004, no medical school in Australia taught statistics and research skills in an integrated way as part of the rest of their problem based learning (PBL) course [32]. As Bland mentions, not only does integration prevent marginalisation of the subject, but it also has the advantage of being taught in the context in which students will use it. Problem based learning has been shown to be an effective way of teaching public health [33] and to be a richer and more engaging approach to the teaching of epidemiology than traditional courses [34]. This principle suggests that the teaching/research exercise could be further improved by integrating it with the rest of the course.

The benefit-to-cost analysis of this type of teaching also needs to consider the additional benefits in the form of scientific outputs (published findings on the health impact of the built environment). There is also potential for policy changes that might contribute to improved student and public health.

The authors agreed with the students' recommendation that the survey be repeated near the end of the academic year after winter 2008 and a repeat survey was conducted in October, spring-time in the Southern Hemisphere. Findings from these surveys have been compared with student union records of the quality of student flats in Dunedin. These results are also being reported back to the University of Otago administration (of which the Medical School is a part) and are forming part of a community-action plan to improve the quality of student flats.

At the conclusion of the class, the tutors asked if anyone wanted to enrol in a paid summer studentship and work with the tutors to analyse the results and write up the teaching evaluations. The first author volunteered and was able to further reinforce her knowledge of research-based teaching and the impact of housing on health.

Our findings have wider implications for teaching of research and public health skills to medical students and other health sciences students. Many Departments such as ours are no doubt using innovative teaching methods and either not evaluating them, or not reporting them in the published literature. To improve evidence-informed teaching practice, it would be useful if other programmes teaching public health and research skills to medical students also published on these activities.

## Conclusion

In this pilot study it was feasible to integrate medical student teaching with real-world research. We found that a large majority of the students responded favourably to the teaching exercise and this was generally successful in raising the profile of public health and research. This approach to integrated teaching/research should be considered further in health sciences training and continue to be evaluated and refined.

## Competing interests

The authors declare that they have no competing interests.

## Authors' contributions

MB and PHC developed and delivered the teaching session being evaluated. MB, PHC, NW and ND helped develop the questionnaire used for assessing medical students housing conditions and respiratory health and contributed to analysis of the findings. ND organised for the distribution of questionnaires to the medical students and their collection and data entry. MB, PHC and ND designed the teaching evaluation. EM undertook literature searches and drafted the paper. All authors reviewed successive drafts of this paper and read and approved the final manuscript.

## Pre-publication history

The pre-publication history for this paper can be accessed here:



## References

[B1] Rego PM, Dick ML (2005). Teaching and learning population and preventive health: challenges for modern medical curricula. Med Educ.

[B2] Bellas PA, Asch SM, Wilkes M (2000). What students bring to medical school: attitudes toward health promotion and prevention. Am J Prev Med.

[B3] Eggert RW, Parkinson MD (1994). Preventive medicine and health system reform. Improving physician education, training, and practice. JAMA.

[B4] Rosenthal O (1998). Changes in Medical education: the beliefs of medical students. Med Educ.

[B5] Wolf TM, Balson PM, Faucett JM, Randall HM (1989). A retrospective study of attitude change during medical education. Med Educ.

[B6] Dare AJ, Bullen C (2008). Shifting perceptions and challenging the profession's paradigms: reflections from an undergraduate week of population health. N Z Med J.

[B7] Linz W, Hoban JD (1992). The need to foster students' research skills. Acad Med.

[B8] Davie GS, Baker MG, Hales S, Carlin JB (2007). Trends and determinants of excess winter mortality in New Zealand: 1980 to 2000. BMC Public Health.

[B9] Schluter PJ, Macey PM, Ford RP (2000). The relationship between inside and outside ambient temperatures in Christchurch, New Zealand. Paediatr Perinat Epidemiol.

[B10] Howden-Chapman P, Saville-Smith K, Crane J, Wilson N (2005). Risk factors for mold in housing: a national survey. Indoor Air.

[B11] Howden-Chapman P, Matheson A, Crane J, Viggers H, Cunningham M, Blakely T, Cunningham C, Woodward A (2007). Effect of insulating existing houses on health inequality: cluster randomised study in the community. BMJ.

[B12] Douwes J, Siebers R, Wouters I, Doekes G, Fitzharris P, Crane J (2006). Endotoxin, (1 --> 3)-beta-D-glucans and fungal extra-cellular polysaccharides in New Zealand homes: a pilot study. Ann Agric Environ Med.

[B13] Purvis DJ, Thompson JM, Clark PM, Robinson E, Black PN, Wild CJ, Mitchell EA (2005). Risk factors for atopic dermatitis in New Zealand children at 3.5 years of age. Br J Dermatol.

[B14] Butler S, Williams M, Tukuitonga C, Paterson J (2003). Problems with damp and cold housing among Pacific families in New Zealand. N Z Med J.

[B15] Shannon S, Lloyd B, Roos J, Kohlmeyer J (2003). EVH3 – Impact of Housing on Health in Dunedin NZ.

[B16] Howden-Chapman P, Crane J, Matheson A, Viggers H, Cunningham M, Blakely T, O'Dea D, Cunningham C (2005). Retrofitting houses with insulation to reduce health inequalities: aims and methods of a clustered, randomised community-based trial. Soc Sci Med.

[B17] Holst PE, Coleman ED, Sheridan JE, O'Donnell TV, Sutthoff PT (1983). Asthma and fungi in the home. N Z Med J.

[B18] Zock JP, Jarvis D, Luczynska C, Sunyer J, Burney P (2002). Housing characteristics, reported mold exposure, and asthma in the European Community Respiratory Health Survey. J Allergy Clin Immunol.

[B19] Howden-Chapman P, Pierse N, Nicholls S, Gillespie-Bennett J, Viggers H, Cunningham M, Phipps R, Boulic M (2008). Effects of improved home heating on asthma in community dwelling children: randomised controlled trial. BMJ.

[B20] Baker M, McNicholas A, Garrett N, Jones N, Stewart J, Koberstein V, Lennon D (2000). Household crowding a major risk factor for epidemic meningococcal disease in Auckland children. Pediatr Infect Dis J.

[B21] Keall M, Baker M, Howden-Chapman P, Cunningham M (2008). Association between the number of home injury hazards and home injury. Accid Anal Prev.

[B22] Smith FG, Harasym PH, Mandin H, Lorscheider FL (2001). Development and evaluation of a Research Project Program for medical students at the University of Calgary Faculty of Medicine. Acad Med.

[B23] Harrison A (1997). Incorporating human subject research experience early in the medical curriculum. Med Teach.

[B24] Freeman J, Dobbie A (2005). Teaching medical students research while reaching the underserved. Fam Med.

[B25] Ax S, Kincade E (2001). Nursing students' perceptions of research: usefulness, implementation and training. J Adv Nurs.

[B26] Retsas A, Nolan M (1999). Barriers to nurses' use of research: an Australian hospital study. Int J Nurs Stud.

[B27] Robert M (1997). What do registered nurses and midwives fell and know about research?. J Adv Nurs.

[B28] El Ansari W (2004). Appraisal skills as a public health competency for evidence-based care: students' satisfaction with their research education. J Public Health Manag Pract.

[B29] Asher MI, Keil U, Anderson HR, Beasley R, Crane J, Martinez F, Mitchell EA, Pearce N (1995). International Study of Asthma and Allergies in Childhood (ISAAC): rationale and methods. Eur Respir J.

[B30] Baker M, Keall M, Au EL, Howden-Chapman P (2007). Home is where the heart is – most of the time. N Z Med J.

[B31] Harvey S (2008). Ill-health blamed on mouldy flat. Otago Daily Times.

[B32] Bland JM (2004). Teaching statistics to medical students using problem-based learning: the Australian experience. BMC Med Educ.

[B33] Gurpinar E, Musal B, Aksakoglu G, Ucku R (2005). Comparison of knowledge scores of medical students in problem-based learning and traditional curriculum on public health topics. BMC Med Educ.

[B34] Dyke P, Jamrozik K, Plant AJ (2001). A randomized trial of a problem-based learning approach for teaching epidemiology. Acad Med.

